# Natural Polyphenols and Spinal Cord Injury

**DOI:** 10.6091/ibj.1278.2014

**Published:** 2014-07

**Authors:** Ali Reza Khalatbary

**Affiliations:** *Molecular and Cell BiologyResearchCenter, Faculty of Medicine, MazandaranUniversity of Medical Sciences, 18KM Khazar Blvd, Khazar Sq. Sari, Iran*

**Keywords:** Spinal cord injury (SCI), Polyphenols, Antioxidants, Herbal medicine

## Abstract

Polyphenols have been shown to have some of the neuroprotective effects against neurodegenerative diseases. These effects are attributed to a variety of biological activities, including free radical scavenging/antioxidant and anti-inflammatory and anti-apoptotic activities. In this regard, many efforts have been made to study the effects of various well-known dietary polyphenols on spinal cord injury (SCI) and to explore the mechanisms behind the neuroprotective effects. The aim of this paper is to present the mechanisms of neuroprotection of natural polyphenols used in animal models of SCI.

## INTRODUCTION

Spinal cord injury (SCI) is a complicated multifactorial process that is caused initially by mechanical trauma and then by diverse mechanisms of secondary injury [1]. The outcome of SCI depends on the extent of secondary damage mediated by a series of cellular, molecular, and biochemical cascades, including calcium ion influx [2], oxygen free radical-induced lipid peroxidation [3, 4], inflammatory reaction [5], autoimmune response [6], vascular events [7], and apoptosis [8]. In recent years, much attention has been focused on secondary injury, because it appears to be susceptible to therapeutic interventions that may include the use of anti-apoptotic, free radical scavenger, and anti-inflamm-atory agents. Polyphenols, one of the most numerous and ubiquitous groups of plant metabolites, are natural compounds that exert a variety of biological actions such as antioxidant, anti-inflammatory, and anti-proliferative activities [9, 10]. The main sources of these molecules are plants and fruits. Based on the molecular structure, polyphenols are classified into flavonoids such as flavonols and isoflavonoids and non-flavonoids such as saponin [11]. These components have an aromatic ring with one or more hydroxyl group(s) [12]. Within the previous decades, a rapidly growing number of natural polyphenol compounds, both flavonoids and non-flavonoids, with neuroprotective effects against neurodegenerative diseases have been described [13]. Olive oil [14], green tea [15], turmeric [16], and grape [17] are the best known of the resources. In this review, we have focused on neuroprotective effects of various well-known dietary polyphenols on SCI and their molecular mechanisms responsible for the neuroprotection (Table 1). 

***Green tea polyphenols in SCI. ***The chemical composition of green tea contains many polyphenolic compounds. These polyphenols, generally known as catechins, consist of eight types of flavonoids [15]. In regard to bioavailability of catechins, it has been shown that these components are detectable in plasma and urine after consumption of green tea [18]. Catechins have many biological actions such as free radical scavenging/antioxidant actions, preventing lipid peroxidation due to oxidative stress, modulating apoptotic pathways, pro-oxidant properties, and anti-inflammatory effects [15]. The first requirement for a dietary compound to be a potential *in vivo* antioxidant is that it enters the blood circulation. In this regard, it is well documented that catechins are able to cross the blood-brain barrier in relatively large amounts, which may account for its neuroprotective properties [15]. Epigallocathechin-gallate (EGCG), the most abundant composition of the tea catechins, has been shown to have some of the protective effects against neuronal damage after transient ischemia [19], oxidative damage on periventricular white matter in hydrocephalic rats [20], suppression of disease progression of amyo-trophic lateral sclerosis [21], acute hypoxia [22], iron-induced oxidative stress [23], Alzheimer's and Parkinson's diseases [24], aging [25], and neuropatic pain [26]. Experimental studies have shown that treatment of SCI with EGCG [27-29] and green tea extract [30] attenuates neuronal apoptosis, spinal tissue loss, lipid peroxidation, inflammatory response, and motor dysfunction. Apoptosis is a key mechanism of secondary damages after SCI [31] that is regulated by the Bcl-2 family proteins, and this represents a potentially avoidable event by pharmacological interventions. Results of immunohistochemical assess-ment showed that the treatment with EGCG reduced positive staining for Bax, while on the contrary, it increased positive staining for Bcl-2 in the EGCG treatment groups after traumatic SCI. These results provide the molecular evidence for the neuroprotective activity of EGCG [27, 28]. Paterniti *et al.* [30] found that green tea extract treatment (1 and 6 hours after spinal cord trauma induction) prevented the SCI-induced Bax expression and significantly reduced the SCI-induced inhibition of Bcl-2 expression. In this regard, *in vitro* and *in vivo* studu suggested the protective effects of EGCG on neural apoptosis via altering the expression of anti-apoptotic and pro-apoptotic genes [15]. EGCG suppressed apoptosis induced by oxidative radical stress through increasing phosphatidylinositol-3 kinase/Akt-dependent anti-apoptotic signals [32]. Moreover, recent studies have shown that EGCG inhibits caspase 3 activation in the spinal cord in amyotrophic lateral sclerosis model mice [21] and in aging mice that was induced by D-galactose [25]. Other study showed that EGCG prevented Bax and Bad expression, while it induced Bcl-2 to protect SHSY5Y cells from apoptosis [33]. Lipid peroxidation is an important pathologic event in post-traumatic neuronal degeneration and reaches to peak values immediately after SCI [34]. Thus, inhibition of lipid peroxidation is thought to be one of the principal mechanisms of action for therapeutic agents. Biochemical studies showed that administration of EGCG immediately and one hour after traumatic SCI significantly attenuated the level of malondialdehyde (MDA), as a product of lipid peroxidation, compared to those of trauma group [28]. Also, Paterniti *et al.* [30] found that the green tea extract treatment caused a significant reduction of the MDA levels in the injured tissue. Green tea polyphenols (mainly EGCG), due to the hydroxyl groups, can bind to the free radicals and neutralize them [15]. Moreover, they can indirectly increase the body's endogenous antioxidants [35]. Previous studies reported that lipid peroxidation was reduced by EGCG administration after cerebral ischemia in rats [19] and gerbils [36]. Recently, some investigators have revealed that MDA levels are decreased after EGCG treatment on aging mice model [25]. 

**Tabel 1:** Studies that have investigated the neuroprotective effects of natural polyphenols using experimental models of SCI

**Polyphenols**	**Model of SCI**	**Effects**	**Reference**
Green tea	Trauma	Bax, Bcl-2 MDA MPO, demyelination TNF-α, IL-1β, iNOS, COX-2, PARP, nitrotyrosine	[27, 28, 30] [28, 30] [29] [29, 30]
			
Olive oil	Trauma	Lipid peroxidation Bax, Bcl-2, GSH TNF-α, IL-1β, iNOS, COX-2, PARP, nitrotyrosine FAS, Caspase-3, GDNF, IKK-α, NF-κβ P65	[61, 63, 64] [61] [62, 64] [64]
			
Resveratrol	Trauma I/R	Edema, MDA MDA, GSH, xanthine oxidase TNF-α, IL-1β, IL-10, MPO, Oxidative stress, NO Oxidative stress, neutrophil infiltration	[92] [93] [94] [95] [96]
			
Turmeric	Trauma Hemisection	Lipid peroxidation Glutathione peroxidase Superoxidase dismutase, catalase Astrocyte reaction Astrocyte reaction	[114, 116, 117] [109] [114, 116] [118, 119] [113]

Malondialdehyde (MDA), Myeloperoxidase (MPO), Isoform of nitric oxide synthase (iNOS), Poly (ADP-ribose) polymerase (PARP), Glial cell-derived neurotrophic factor (GDNF), Glutathione (GSH), Ischemia-reperfusion (I/R)

Although the most important properties of catechins have long been attributed to the antioxidant and free radical scavenging effects, emerging evidences have shown the neuroprotective effects of the catechins against neurodegenerative or neuroinflammatory diseases [24, 37]. Injury to the spinal cord provokes local inflammatory response involved in non-cellular and cellular components, which amplifies the secondary damage. In this regard, biochemical study showed that tissue myeloperoxidase activity, an indicator of neutrophil infiltration, was significantly decreased in EGCG-treatment groups after traumatic SCI [29]. Some evidences suggested that these cells play an important role in the pathogenesis of secondary degeneration such as lipid peroxidation and myelin vesiculation [5]. Moreover, a reduction in demy-elination was observed in EGCG treatment groups [29]. It is well documented that the potent pro-inflammatory cytokines (including TNF-α and IL-1β) which are synthesized immediately after injury, nitrotyrosine, isoform of nitric oxide synthase (iNOS), COX-2, and poly (ADP-ribose) polymerase (PARP), play detrimental roles in post-traumatic injury associated with SCI [38, 39]. In another study, attenuated TNF-α, IL-1β, nitrotyrosine, iNOS, COX-2, and PARP expression was detected in the EGCG-treated rats after traumatic SCI [29]. Meanwhile, similar results were documented by Paterniti *et al.* [30]. There is substantial evidence that the anti-inflammatory effects of EGCG, the most effective catechin, may be due in part to the inhibition of iNOS [15]. In this regard, *in vitro *study indicated that EGCG inhibited the induction of iNOS mRNA after treatment with TNF-α and IL-1 [40]. Moreover, it has been well established that EGCG inhibits iNOS activity and expression following brain damage [41]. Catechins also enhanced the production of IL-10, an anti-inflammatory cytokine [42]. Another study revealed that the production of eicosanoids by COX, a major pathway leading to the endpoint of inflammation, significantly reduced post-ischemia by catechins [43]. It has been documented that catechins can scavenge peroxynitrite by preventing tyrosine nitration [44]. 

***Olive oil polyphenols in spinal cord injury. ***Olive oil is a source of at least 30 phenolic compounds with either one or two hydroxyl group(s). This oil is divided into three categories: secoiridoids such as oleuropein (3,4-dihydroxyphenylelenolic acid) and oleocanthal, simple phenols such as hydroxytyrosol (3,4-dihydroxyphenolethanol) and tyrosol (4-hydroxyphenylethanol), and lignans [45, 46]. Animal and human studies have demonstrated that olive oil polyphenols are highly bioavailable. In this regard, one study showed that apparent *in vivo* absorption of the ingested olive oil polyphenols was more than 55-66 mol% in humans [47]. There is accumulating evidence that attributed the beneficial effects of olive oil phenols to a variety of biological activities, including free radical scavenging/antioxidant, anti-inflammatory, anti-carcinogenic, anti-microbial, anti-atherogenic, and antiviral properties [48, 49]. In addition, olive oil phenols have been shown to have some of the neuroprotective effects against brain hypoxia-reoxigenation [50, 51], cerebral ischemia [52, 53], and brain damage after hypoxia-reoxygenation in diabetic rats [54], ageing [55], Alzheimer’s diseases [56], Huntington’s disease [57], multiple sclerosis [58], Parkinson’s disease [59], peripheral neuropathy [60], and SCI [61-64]. It is well documented that olive oil polyphenols are able to cross the blood-brain barrier [65]. Previous results showed that administration of oleuropein immediately and one hour after traumatic SCI significantly attenuated lipid peroxidation compared to that in trauma group [61]. Meanwhile, oleuropein attenuated somewhat myelin degradation in the site of contusion [61]. Also, Impellizzeri *et al.* [64] documented that oleuropein aglycone, a hydrolysis product obtained from oleuropein, in a mice model of spinal cord trauma significantly decreased lipid peroxidation. One of the neuroprotective mechanisms of dietary virgin olive oil and its phenolics in hypoxia-reoxygenation and transient focal cerebral ischemia may be due in part to its effects on free radical-induced lipid peroxidation [66-69]. Olive oil reduced tissue lipid peroxidation by 20.3% in brain in hyperlipemic rabbits [70]. A recent study has shown that extra virgin olive oil and hydroxytyrosol exert strong antioxidative effects on a 3NP-induced Huntington’s disease-like rat model by reducing lipid peroxidation product levels, blocking glutathione depletion, and blocking and reversing the effect of 3NP on succinate dehydrogenase activity [71]. Glutathione has been found to display potent antioxidant properties. It has been well known that promotion of glutathione synthesis after SCI would be an effective way to reduce oxidative stress, tissue damage, and motor disfunction [72]. Biochemical study showed that administration of oleuropein after traumatic SCI significantly increased the level of glutathione [61]. In support of these findings, other study documented that dietary olive oil increased glutathione concentration in rat brain slices subjected to hypoxia-reoxygenation [66]. Also, oleuropein increased the expression of glutathione-related enzymes at transcriptional level [73]. Results of immunohistochemical assessment showed that the treatment with oleuropein reduced positive staining for Bax, while on the contrary, it increased positive staining for Bcl-2 in the oleuropein treatment groups after spinal cord trauma [61]. Also, Impellizzeri *et al.* [64] documented that oleuropein aglycone significantly decreased FAS ligand, caspase 3, and Bax expression after spinal cord trauma. A finding in the model of brain hypoxia-reoxygenation showed that in rats treated with olive oil, brain cell death was 42.5% lower than in untreated rats [66]. In explaining this finding, it was documented that olive oil modulates the inducible iNOS in brain tissues [66]. A further study showed that oral administration of olive oil reduced infarct volume in rats subjected to ischemia-reperfusion [53]. 

Some studies have documented that oleuropein elicits anti-inflammatory effects by lypoxygenase activity and the production of leukotriene B_4 _[74], inhibiting biosynthesis of pro-inflammatory cytokines [75, 76], or modulating inflammatory parameters [77]. Immunohistochemical studies demonstrated that oleuropein treatment significantly attenuated the expression of TNF-α and IL-1β, and consequently expression of iNOS and COX-2 after traumatic SCI [62]. Meanwhile, pro-inflammatory cytokine production (such as TNF-α and IL-1β) and iNOS expression were significantly decreased after administration of oleuropein aglycone in spinal cord trauma [64]. It has been well established that olive phenolics inhibit iNOS activity or the inflammatory mediators that stimulate this enzyme following brain hypoxia-reoxygenation [66]. Olive oil phenolic compounds decrease the circulating concentrations of IL-6, a pro-inflammatory agent that stimulates inflammation in response to trauma [78]. Another study has also shown that the olive oil phenolic compounds inhibit COX-2 activity [79]. Impellizzeri *et al.* [76] reported that the administration of oleuropein in a mouse model of carrageenan-induced pleurisy caused a significant reduction in TNF-α, IL-1β, and nitric oxide [76]. Also, it was demonstrated that oleuropein treatment significantly attenuated expression of PARP and nitrotyrosine after spinal cord trauma [62]. In this regard, an investigation has shown that olive oil polyphenols significantly reduce peroxynitrite formation [80]. Another study documented that administration of oleuropein attenuated nitrotyrosine and PARP [76]. Also, it was observed that the myeloperoxidase activity was reduced significantly in oleuropein-treated rats when compared with non-treated rats after traumatic SCI [63]. In this regard, it has been documented that oleuropein strongly inhibited the enzyme myeloperoxidase in the inflamed tissue [81]. Impellizzeri *et al.* [64] documented a similar result after oleuropein aglycone treatment in an experimental model of SCI in mice. Visioli and colleagues [82] reported that oleuropein inhibits the respiratory burst of neutrophils and hypochlorous acid-derived radicals. Moreover, other study showed that olive oil polyphenols inhibited endothelial-leukocyte adhesion molecule expression [83]. Oleuropein aglycone treatment significantly increased glial cell-derived neurotrophic factor levels after spinal cord trauma in mice [64]. This treatment has a potent survival-promoting effect on various neuronal populations. Also, oleuropein aglycone administration prevented SCI-induced IkB-α degradation, reduced the levels of IKK-α and NF-kB p65 and restored protein kinase A levels in SCI-operated mice [64].

***Grape polyphenols in*** ***spinal cord injury*** ***. ***Resveratrol (3,4^´^,5-trihydroxy-trans-stilbene) is a natural non-flavenoid polyphenol present in many plants including grapes [84]. Resveratrol has been reported to have a large number of pharmacological properties, including anti-cancer, antioxidant, cardioprotective, and anti-inflammatory properties [85, 86]. Several studies have shown that resveratrol could exert neuroptective effects in Alzheimer’s disease [87], Parkinson’s disease [88], traumatic brain injury [89], cerebral ischemia [90], spinal cord trauma [91-94], and spinal cord ischemia [95-97]. Wang and colleagues [98] documented that resveratrol can pass the blood-brain barrier and induce neuroprotective effects. Meanwhile, new drug delivery systems to improve the bioavalability of resveratrol have been developed [99]. In addition, although the mechanisms of the neuroprotective effects of resveratrol are not fully understood, *in vitro* and *in vivo* studies have shown that the neuroroprotective mechanisms of action by resveratrol could be attributed to its antioxidant, anti-inflammatory, and anti-apoptotic properties [86, 87]. 

Studies have shown that resveratrol has a protective role in animal models of SCI. In this regard, Yang and Piao [91] documented that resveratrol (100 and 500 mg/kg immediately after spinal cord trauma, intraperitoneally) protected the spinal cord through improving Ca^2+^, Mg^(2+)^-ATPase system. They also reported, for the first time, that resveratrol (50 and 100 mg/kg immediately after spinal cord trauma, intraperitoneally) strongly affected the secondary pathophysiological reaction including spinal cord edema (reduction of 11.5% at 48 h after spinal cord trauma), energy metabolism system such as lactate dehydrogenase activity (suppression of 40% at 48 h after spinal cord trauma) and Na^+^, K^+^-ATPase activity (promotion of 60% at 48 h after spinal cord trauma), and lipid peroxidation (reduction of MDA, 40% at 48 h after after spinal cord trauma). Moreover, resveratrol-treated rats soundly maintained the ultrastructure of the injured spinal cord in the relatively good appearance after traumatic SCI [92]. It has been known that energetic metabolism is seriously disturbed in injured spinal cord tissue [100]. Levels of lactate dehydrogenase and Na^+^, K^+^-ATPase activity are the most important indices of energy metabolism changes, lactate dehydrogenase activity increased, and Na^+^, K^+^-ATPase activity inhibited after injury [101]. Resveratrol, as an antioxidant that prevents lipid peroxidation, has three phenolic hydroxyl groups which can competitively be combined to free radicals and reduce their content *in vivo* [102]. In another study, the resveratrol-induced protection against ischemia-reperfusion injury has been documented in rabbit's spinal cord [95]. In this model of SCI, preischemic infusion of 10 mg/kg resveratrol protected spinal cord from ischemia-reperfusion injury through decreased oxidative stress and increased nitric oxide release [95]. Some reports have indicated that antioxidant properties of resveratrol are explained by its stimulation of nitric oxide formation [103, 104]. In another published study, preischemic infusion of resveratrol (100 µg/kg intravenously) protected the spinal cord from ischemia-reperfusion injury in rabbits due to the decreasion of oxidative stress and neutrophil infiltration, and probably due to promotion of collateral blood flow to the ischemic spinal cord segments [96]. Meanwhile, neurologic impairment was significantly lower in the resveratrol-treated animals [96]. Ates *et al.* [93] demonstrated that resveratrol treatment (100 mg/kg immediately after traumatic SCI, intraperiteonally) significantly decreased malodialdehyde, nitric oxide, xanthine oxidase and increased glutathione levels than methylprenisolone treatment. Accumulated studies have shown that resveratrol has anti-inflammatory and anti-apoptotic effects [105-107]. In this regared, Liu *et al.* [94] have recently documented that the expression of inflammatory cytokines including IL-1β, IL-10, TNF-α and myeloperoxidase is suppressed by resveratrol (200 mg/kg, i.p., three times per day for 3 days) after spinal cord trauma. In this study, it has also been shown that resveratrol treatment affects the expression level of apoptosis-related gene Bax, Bcl-2, and caspase 3, indicating its anti-apoptotic properties [94].

***Turmeric polyphenols in*** ***spinal cord injury*** ***. ***Turmeric (Curcuma longa) is a rhizomatous herbaceous perennial plant of the ginger family, Zingiberaceae. The most important chemical components of turmeric are a group of compounds called curcuminoids, which include curcumin, demethoxycurcumin, and bisdemethoxycurcumin. The best studied compound is curcumin, which constitutes 3.14% (on average) of powdered turmeric. Curcumin (diferuloylmethane) [1,7-bis(4-hydroxy-3-methoxy-phenyl)-1,6-heptadiene-3,5-dione] is a polyphenolic, non-flavanoid compound and exhibits a wide range of pharmacological activities, including antioxidant, anti-inflammatory, anti-carcinogenic, anti-bacterial, immunomodulatory, and anti-apoptotic activities [16, 108]. This component possesses the phenolic, β-diketone, and the methoxy groups which contribute to its free radical scavenging properties [16]. On the other hand, it has been documented that curcumin induces endogenous antioxidant defense mechanisms [16]. Curcumin have been shown to have some of the neuroprotective effects against brain trauma [109], cerebral ischemia [110], Parkinson’s disease [111], neuropathic pain [112], and SCI [113, 114]. Although animal and human studies have shown that bioavailability of curcumin is very limited due to low intestinal absorption, rapid metabolism in liver, and elimination through gall bladder [16], it is documented that curcumin is able to permeate the blood-brain barrier and to exert neuroprotection effects [115]. Curcumin treatment improved neurologic outcome after SCI, which was supported by decreased level of lipid peroxydation [114, 116, 117], increased level of glutathione peroxidase activity [109], and attenuated level of apoptosis [113]. Also, it is documented that treatment with 200 mg/kg curcumin increased superoxide dismutase and catalase activity after closing force [114] and weight drop method of SCI [116]. Meanwhile, curcumin attenuated the level of astrocyte reactivation after spinal cord hemisection [113] and impact injury [118]. Ormond *et al.* [119] found that epidural administration of curcumin (60 mg/kg/ml body weight within 30 minutes after contusion and weekly thereafter) significantly improved motor function compared with controls [119]. Also, an increased neural element mass with less gliosis found at the contusion site in curcumin-treated rats than controls. 

***Other polyphenols in*** ***spinal cord injury*** ***. ***Some studies have started evaluating the neuroprotective effect of polyphenols from diverse natural products against SCI. Among these compounds, we can refer to Ginkgo Biolba leaf extract (EGb 761) and ginseng extract. 

EGb 761 contains multiple compounds such as polyphenols that are thought to contribute to its neuroprotective properties [120]. In this regard, some studies documented that EGb 761 can inhibit nerve cell apoptosis [121, 122] and lipid peroxidation [122] and scavenge free radicals production [123] in spinal cord after ischemia-reperfusion injury in rabbits. Zhao *et al.* [124] found that EGb 761 protected spinal cord neurons from glutamate excitotoxicity and oxidative stress-induced cell death through inhibition of cytosolic phospholipase A2 activation, an enzyme that is known to play a key role in mediating secondary pathogenesis after acute SCI. Ao and colleagues [125] documented that the apoptotic index and the percentage of iNOS-positive cells were lower in EGb 761 group than in control group after spinal hemisection injury.

Ginseng root extract has been used for the treatment of neurological disorders. Studies have shown that ginseng saponins (ginsenosides), a major component in ginseng root extract, can cross the blood-brain barrier and induce neuroprotection [126]. An* in vitro* study showed that ginsenosides dose-dependently protected spinal cord neurons from death induced by excitotoxicity and oxidative stress [127]. Using the compressive SCI model, it was shown that intravenous infusion of dihydroginsenoside Rb1 improved SCI [128]. In another study, it was found that panax notoginsenoside protected spinal cord after ischemia-reperfusion injury, which is probably mediated by its anti-inflammatory, anti-edema and anti-apoptotic actions [129].

## CONCLUSION

Polyphenols are dietary components that exert a variety of biochemical and pharmacological effects. The bulk of published data illustrates that many natural polyphenols of diet are effective in protecting against neurodegenerative diseases such as SCI. In this review, the most commonly used polyphenols in experimental models of SCI have been discussed. The evidence presented in this review supports the neuroprotective effects of polyphenols, which is mediated by modulating of the complex secondary injury cascade after SCI, including oxygen radical-mediated lipid peroxidation, inflammatory reactions, and apoptosis. Accordingly, these components may constitute an effective means of interrupting secondary cascades after experimental models of SCI. Of course, there are still questions regarding the use of these compounds in neurological damages. One of the important questions to answer is whether the investigated polyphenols reach the neural tissue in sufficient concentrations and in biologically active forms. Generally, there is little information regarding the interaction of polyphenols on the blood-brain barrier. In this regard, production of conjugates can be a good strategy to promote polyphenol absorption and activity. More importantly, safety, hazards, and risks of consuming polyphenols as life-long therapeutics should always be considered. In this regard, there are few reports about adverse effects of polyphenols. For example, high-dose green tea polyphenols in the diet disrupted kidney function through the reduction of antioxidant enzymes [130], and enhanced tumor development in colon [131]. In addition, preclinical and clinical trial researches utilizeing the dietary supplementation to assess its potential in prevention and treatment of SCI remain scarce.
